# Autologous conditioned serum: clinical and functional results using a novel disease modifying agent for the management of knee osteoarthritis

**DOI:** 10.1080/21556660.2020.1734009

**Published:** 2020-03-25

**Authors:** Matteo Vitali, Marco Ometti, Andreas Drossinos, Pierluigi Pironti, Luca Santoleri, Vincenzo Salini

**Affiliations:** a Department of Orthopedics and Traumatology, San Raffaele Hospital Scientific Institute, Milan, Italy; b Immunohematology and Transfusion Medicine, IRRCS Ospedale San Raffaele, Milan, Italy

**Keywords:** Autologous conditioned serum, knee osteoarthritis, disease-modifying osteoarthritic drugs, cytokines, inflammation

## Abstract

**Objective:**

The purpose of this study was to investigate the potential ability of autologous conditioned serum (ACS) to decrease pain and improve joint functionality in patients affected by knee osteoarthritis (OA).

**Methods:**

Fifteen patients with clinical and radiological signs of OA of the knee were recruited for this study. Each patient received 4 injections of ACS (Orthokine; orthogen, Dusseldorf, Germany) at the site of OA once per week for 4 weeks. Clinical and functional evaluation was performed using the VAS scale for pain, WOMAC scale and KSS functional and clinical scores before the first injection, at one week, at two weeks, at three weeks, at one month and at six months. Statistical analysis was done with the Wilcoxon Signed-Rank Test.

**Results:**

Our results show an improvement of all the evaluation scales at 6 months follow-up. Particularly, VAS scales among all patients decreased by 35.8% (*p* = .00148), KSS functional scores improved by 38.2% (*p* = .00148), KSS clinical scores improved by 28.9% (*p* = .00236) and WOMAC scores were reduced by 19.8% (*p* = .00188). Few adverse effects were observed in our sample. The most common complaint was pain and swelling in the subsequent days after performing the intra-articular injection. Only one patient reported rigidity following the injection of the ACS.

**Conclusion:**

Our results, in conjunction with preexisting studies in the medical literature regarding ACS, demonstrate the viability of this therapy for the treatment of knee OA, showing positive influence on pain and joint function without significant adverse effects.

## Introduction

OA is the most common debilitating disease of the musculoskeletal system in adults over the age of 60. To date, there are no definitive treatment options for OA. Current guidelines aim to delay joint replacement surgery as much as possible with the use of conservative treatment modalities^1^. Non-pharmacological options include life-style changes such as weight loss and low impact physical exercises however these tend to have low patient compliance. Of the pharmacological options available, intra-articular injections have proven to be more effective than oral modalities due to their favorable pharmacokinetic properties. Corticosteroid injections were initially proposed to relieve some of the inflammation, although repeated injections are not recommended due to the cartilage degradation that may occur with cortisone^2^. Hyaluronic is another feasible option for patients with mild-to-moderate knee OA with no response to the first-line treatment. It provides mechanical lubrication, facilitating the gliding of the femur over the tibia^3^. In recent years, as the understanding of the mechanisms underlying OA has improved, targeted treatments have been developed to attempt to slow down the progression of this disease. These drugs are called disease-modifying osteoarthritic drugs (DMOADs)^4^.

Indeed, it has been shown that synoviocytes, activated immune cells and chondrocytes secrete cytokines and growth factors that play an important role in cartilage degeneration^5^. Interleukin-1 (IL-1) is such a pro-inflammatory cytokine, suspected to play a prominent role in the pathophysiology of OA^6^^,^^7^. It stimulates matrix metalloproteinases and prostaglandin production, both of which have a negative effect on the cartilage matrix integrity^8^.

DMOADs are believed to intervene with the inflammatory pathways of these cytokines thereby slowing down disease progression, decreasing disease symptoms, and improving quality of life^9^.

Orthokine (Orthogen, Dusseldorf, Germany) is a product designed to stimulate the synthesis of the IL-1 receptor antagonist (IL-1ra) and anti-inflammatory cytokines such as IL-4, IL-10 and IL-13. The product is a serum isolated from whole blood, incubated and then separated with centrifugation. The potentially beneficial effect of Orthokine and other DMOADs on symptoms and progression of OA has been investigated by certain studies in the past decade^10–13^.

The aim of our study was to investigate the effect of Orthokine (ACS) to reduce pain, improve joint function and enhance general quality of life in patients with knee OA using clinical and functional evaluations.

## Materials and methods

Fifteen patients with clinical and radiological signs of OA of the knee were recruited for this study between October 2017 and September 2018 at the San Raffaele Hospital of Milan. In particular, we selected participants that were older than 35, were suffering from knee OA for at least 3 months, and had a visual analog score (VAS) for pain greater than 50 on a 100 mm scale at the time of recruitment. Average age of the cohort was 63.5 years old, although there was significant difference between the genders. The female average was 69 years whereas the males in the study were 57.1 years old on average. Plain radiographs and MRI were performed to assess the grade of OA. Particularly, 7 patients presented a 1st Kallgren-Lawrence (KL) grade, 6 patients a 2nd KL grade and 2 patients a 3rd KL grade. Patients showing a Kellgren-Lawrence grade 5 were excluded from this study. Previous surgery of the studied knee was acceptable provided the intervention occurred more than 12 months prior to the beginning of the intra-articular injections with ACS. In addition, if screening for hepatitis B, hepatitis C and HIV resulted positive, such patients were excluded from the study. Patients were also excluded if they were pregnant or lactating, abused drugs, or had received intra-articular injections of other compounds within the prior 6 months. Joint space width was not measured, as literature indicates that changes may only be apparent radiographically in longer studies of 18–24 months duration. The 6-month follow-up period of the current trial was therefore too short to reasonably expect protective effects to be detected in knee radiographs^14–16^.

All participants underwent a visitation with an orthopedic specialist who provided information regarding the trial, alternative conservative treatment, procedures of the treatment and associated risks.

In patients that were deemed to be admissible to participate in the trial, 50 mL of whole blood were taken using a special syringe containing CrSO_4_-treated grade glass beads in order to promote IL-1ra synthesis and accumulation (Orthogen, Düsseldorf, Germany)^10^^,^^17^. The incubation period lasted 7 h after which, the blood-filled syringes were centrifuged and the serum supernatant was filtered and aliquoted into four 3 mL portions. Since the injections were not performed immediately after the serum preparation, the aliquots were stored at −20 °C until their use was necessary.

Patients received 1 intra-articular injection for 4 consecutive weeks at the site of OA. Knee injections were performed manually without the use of ultrasonography with the patient in a supine position. The site of injection was the superolateral margin of the patella of the affected knee.

Prior to each injection and at each follow up, the patients filled out a VAS for pain, ranging from 0 to 10, and Western Ontario McMaster University (WOMAC) in VAS format to evaluate subjective aspects of the OA on their lives. The WOMAC is a standardized questionnaire that attempts to gather quantitative information regarding arthritic joints of the patient. It is a series of 24 questions that address pain, stiffness, and physical function each graded along a scale ranging from 0 to 100^18^. After these, the orthopedic surgeon performed an objective evaluation of the knee using the Knee Society Score (KSS). The KSS is divided into a clinical part and a functional part. The objective knee score, completed by the surgeon, includes a VAS score of pain walking on level ground and on stairs or inclines, as well as an assessment of alignment, ligament stability, and range of motion, along with deductions for flexion contracture or extensor lag^19^^,^^20^.

Follow-up visits were done at 1 month and at 6 months from the initial intra-articular injection. In total, each individual patient was assessed by an orthopedic surgeon both clinically and functionally, 6 times. At each visit, the patients were evaluated using VAS for pain, WOMAC, and KSS questionnaires.

Due to the lack of control group, a Wilcoxon signed-rank test was performed to evaluate the difference between the values obtained from the subjective and objective assessments of each patient along predetermined time frames using a significance level of *p* ≤ .05.

This study was conducted following the principles of the Declaration of Helsinki and with the patients’ permission expressed through a written consent.

## Results

### Vas

**Figure 1.**
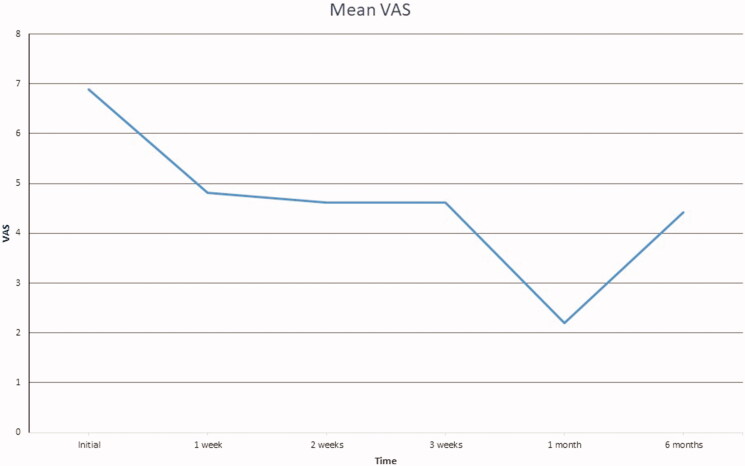
Mean VAS graph at each evaluation.

Mean progression of VAS scores for pain are presented in Figure 1. Wilcoxon signed-rank test analysis confirmed that this change was statistically significant at *p* ≤ .05, as the calculated *p*-value is .00148.

**Figure 2.**
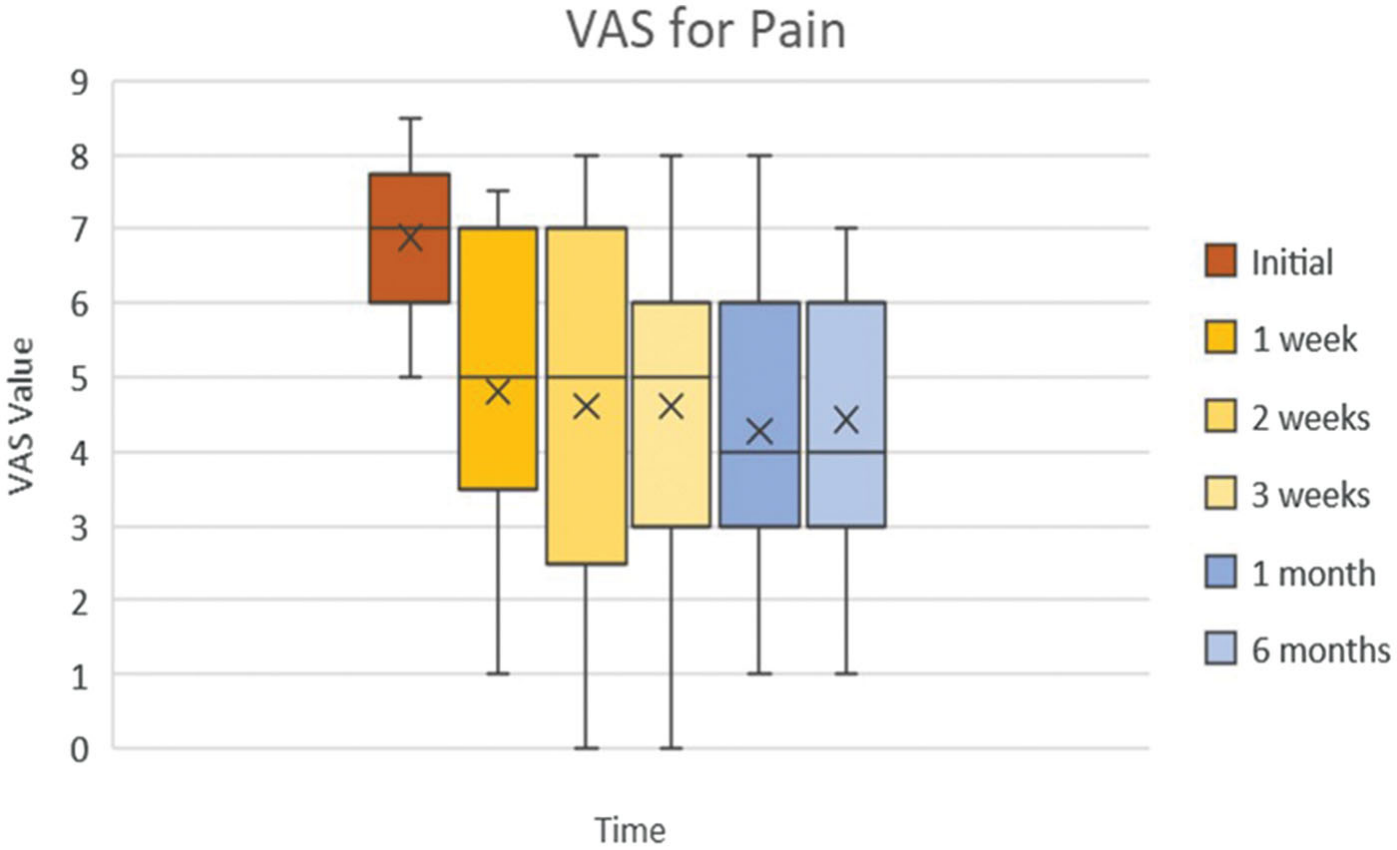
Box plot of VAS according to each clinical evaluation. Whiskers: full range of patients; box: interquartile range (25–75% of patients); X: average; horizontal line within box (if present): median.

Furthermore, the box plot of Figure 2 demonstrates the interquartile range of the VAS questionnaire values at each of the clinical evaluations. Again, there is a general trend toward a declining average VAS score beginning from the initial VAS score measured. The VAS initial displays a more concise interquartile range, as should be expected from our exclusion criteria of requiring at least 5/10 self-reported VAS pain score. However, the box plot demonstrates that there is an increase in the distribution of reported pain scores after the initial assessment. This is seen by the greater amplitude of the boxes starting from the 1st week. There are no outliers present in the chart as all the distributions of the VAS values lie within the interquartile range.

### KSS functional score

**Figure 3.**
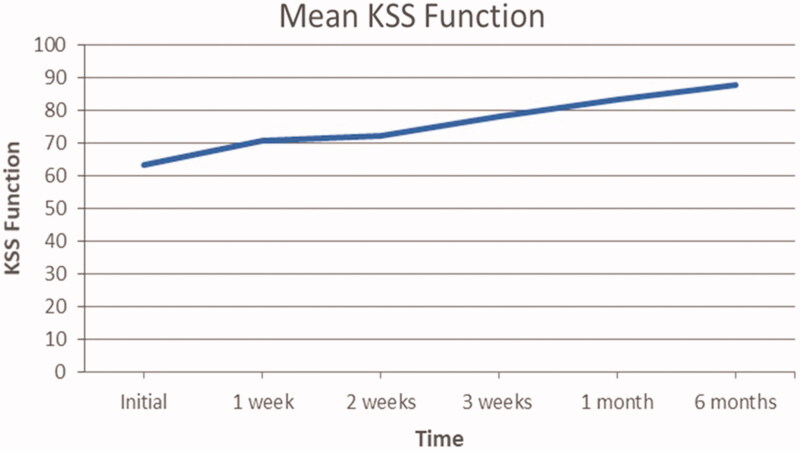
Mean patient KSS functional scores at each clinical evaluation.

Mean progression of KSS clinical scores are presented in Figure 3. The general trend appears to increase over time. In fact, the average population KSS functional scores improved by 38.2% at 6 months (from 63.4 to 87.7) from the initial assessment to the 6 months follow up. Wilcoxon sign-rank test analysis confirms that this change was statistically significant at *p* ≤ .05, as the calculated *p*-value is .00148.

**Figure 4.**
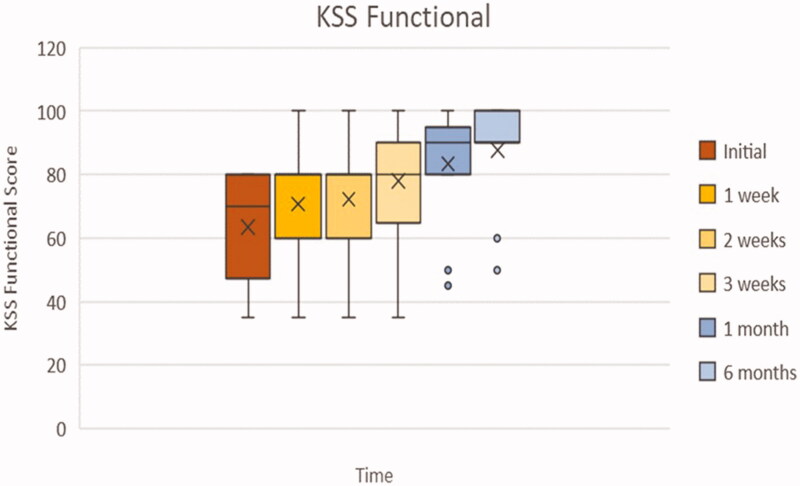
Box plot of KSS functional according to each clinical evaluation. Whiskers: full range of patients; box: interquartile range (25–75% of patients); X: average; horizontal line within box (if present): median.

Figure 4 displays the interquartile ranges of each KSS functional score according to the time at which they were gathered. Again, there is a general trend toward an increase in KSS functional scores. Furthermore, the interquartile range boxes tend to decrease in amplitude with each sequential time plot. This suggests the distribution of values diminishes as time goes by, indicating a uniform positive response to treatment amongst the patients. However, it must be noted that in the follow-up period we can observe 2 distinct data sets that lie well below the lower quartile range of their respective box. These points indicate the presence of 2 patients that at both follow up visits had worsening of KSS functional scores.

### KSS clinical score

**Figure 5.**
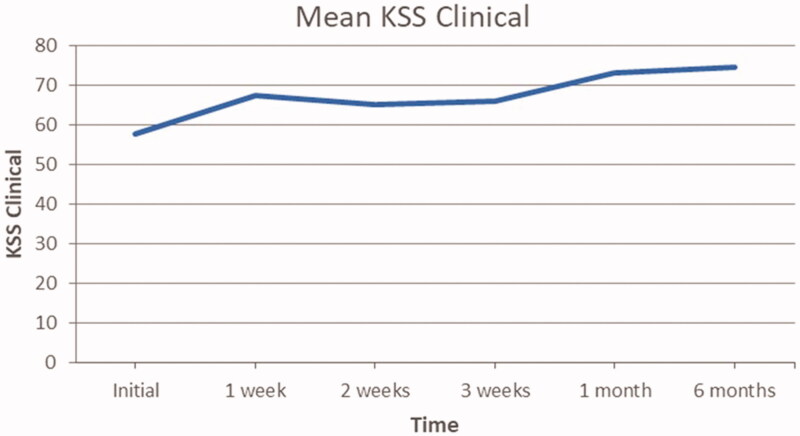
Mean patient KSS clinical scores at each evaluation.

Mean progression of KSS clinical scores are presented in Figure 5. The general trend appears to increase over time. In fact, the average population KSS clinical scores improved by 28.9% (from 57.8 to 74.5) from the initial assessment to the 6 months follow up. Wilcoxon sign-rank test analysis confirms that this change was statistically significant at *p* ≤ .05, as the calculated *p*-value is .00236.

**Figure 6.**
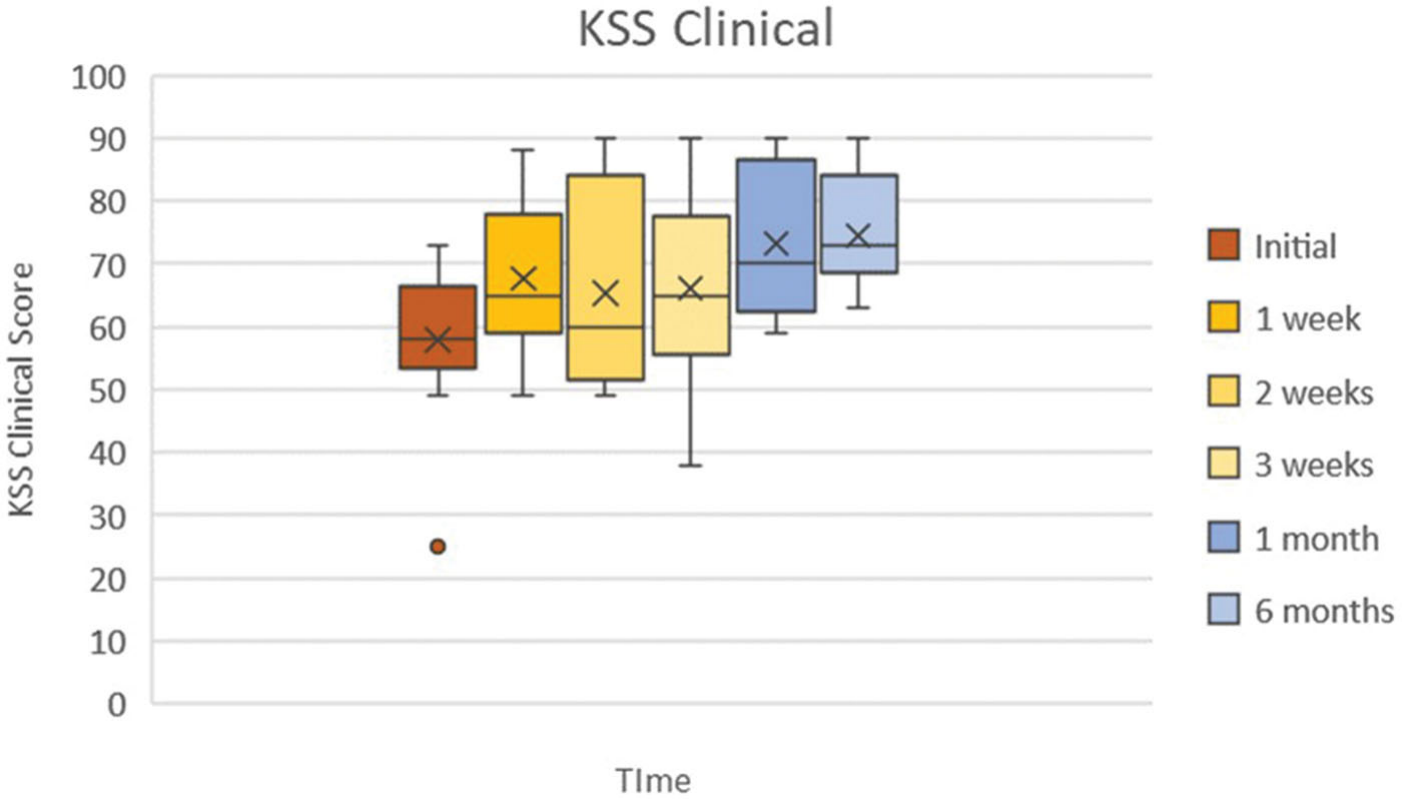
Box plot of KSS clinical scores at each clinical evaluation. Whiskers: full range of patients; box: interquartile range (25–75% of patients); X: average; horizontal line within box (if present): median.

Figure 6 displays the interquartile ranges of each KSS clinical score according to the time at which they were gathered. As reported previously by the graph in Figure 5, there is a general trend toward an improvement of the KSS clinical scores. The box plot demonstrates that the interquartile range varies in amplitude with each sequential time plot and therefore there isn’t a uniform response to the treatment. Although the KSS clinical initial score distribution appears less varied than the other 5 time plots, there is an outlier. This outlier could be explained by a patient starting with substantially poor initial KSS clinical scores as determined by the orthopedic surgeon. The amplitude of the interquartile boxes is greatest at 2 weeks since the initial visit, indicating a wider distribution of response to the therapy at this point. In the follow-up period, specifically the 6 months box, we can see a return to diminished amplitude, indicating congruency of KSS clinical values at 6 months follow up among the patient population.

### Womac

**Figure 7.**
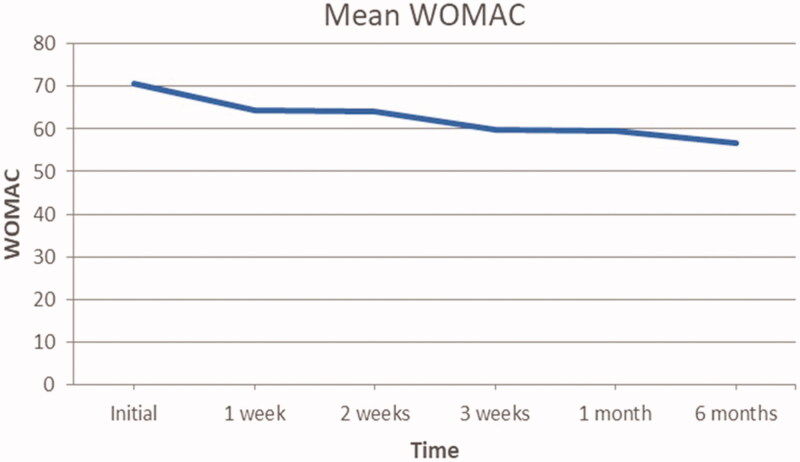
Mean patient WOMAC scores at each evaluation.

Average progression of WOMAC scores are presented in Figure 7. The general trend appears to decrease over time. In particular, the average population WOMAC scores reduced by 19.8% at 6 months follow up from initial scores (from 70.7 to 56.7). Wilcoxon sign-rank test analysis confirms that this change was statistically significant at *p* ≤ .05, as the calculated *p*-value is .00188.

**Figure 8.**
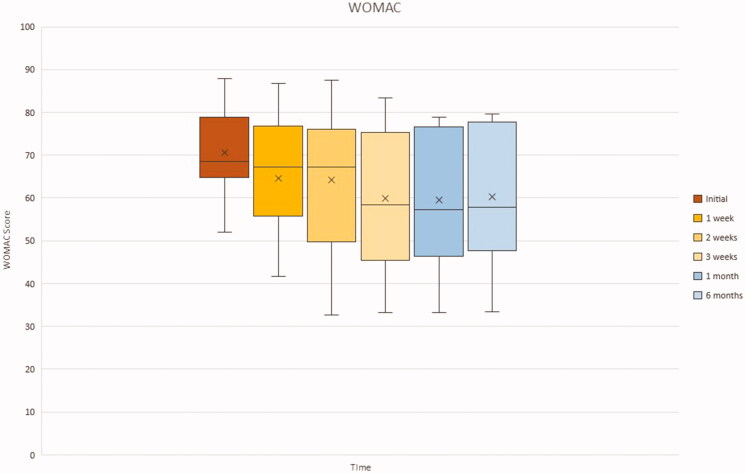
Box plot of WOMAC at each clinical evaluation. Whiskers: full range of patients; box: interquartile range (25–75% of patients); X: average; horizontal line within box (if present): median.

Figure 8 displays the WOMAC scores according to the time at which they were gathered. As seen by the average marked within the box plot, there is a general trend for the WOMAC score to decrease. However, the interquartile range boxes increase in amplitude with each sequential time plot, particularly within the first 4 time plots. The initial time point distribution appears less varied than the other 5 time plots, due to its small size. As time goes by, the range of WOMAC scores increases between the patient population and we can observe an increase in the amplitude of the interquartile boxes, indicating good but varied response to the treatment.

### p-Values

In Table 1, we can see the *p*-values of the comparisons between pre-injection values vs final injection values, pre-injection values vs 6 months follow-up, and final injection values vs 6 months follow-up values of VAS, WOMAC and KSS functional and clinical scores. Significance was set at *p* ≤ .05. The bold values indicate statistically significant values. As we can see, all but two values proved to be statistically significant. Interestingly, these appear in the comparisons between the final injection and the 6 months follow-up of the subjective VAS and WOMAC scores. This indicates that there was no patient-reported significant improvement in knee joint function and pain between the final injection and the 6 months follow-up. However, if we look at the two physician reported scores, the KSS functional and clinical, we can see that the reported *p*-values are statistically significant. This implies that from an objective clinical perspective, there was significant improvement during the time frame of the final injection and the 6 months follow-up. Overall, the most important values to look at are the 2nd column, which show comparisons between the baseline pretreatment scores and the final reported scores taken at the end of the study. This column shows the net effect of the ACS injections on knee OA symptomatology, which in all evaluation scales indicate a statistically significant improvement (*p* ≤ .05) in the knee joint.

**Table 1.** Wilcoxon signed-rank test *p*-values.

	Initial vs 4th injection	Initial vs 6 months Follow-Up	4th injection vs 6 months Follow-Up
VAS	**0.001**	**0.0002**	0.375
WOMAC	**0.0002**	**0.0005**	0.8792
KSS Functional	**0.002**	**0.0002**	**0.0039**
KSS Clinical	**0.0449**	**0.0007**	**0.0015**

Bold values indicate statistically significant values.

### Adverse effects

No significant adverse effects were reported during the duration of the study. In fact, the adverse effects that were reported were due to the physical introduction of the supernatant in the knee joint during the first 4 weeks. Of these, the most common complaint was pain in the subsequent days after performing the intra-articular injection. Five patients reported this peri-injection pain. Three patients reported significant swelling in the immediate days following injection. One patient reported rigidity following the injection of the ACS.

## Discussion

OA is a complex disease involving the entire synovial joint. Although the causes are not fully understood, certain risk factors such as previous injury or gender may induce a predisposition for the development of knee OA^21–25^. To date there are no definitive curative treatment options for OA. Current guidelines aim to first attempt conservative treatment *via* non-pharmacological and/or pharmacological means^2^^,^^26–34^. If these fail to demonstrate a benefit, surgical care is considered^35–39^.

More recently, as the factors responsible for the development of OA become better understood *via* detailed *in vitro* studies, new treatment modalities have been developed for articular cartilage defects. These are the so-called DMOADs^4^. Specifically, the molecular compounds that contribute to the development of OA are now being viewed as potential drug targets. Of these, IL-1 appears to be a major player in the chronic degeneration of the knee joint^6–8^. As such, any potential antagonists of the IL-1 receptor offer promising results in the slowing of OA evolution.

Compared to current synthetic IL-1 receptor antagonists (IL-1 Ra) such as anakinra, ACS is particularly interesting since it is derived from the patient’s own blood^40^^,^^41^. This feature provides an excellent safety profile that minimizes adverse effects and minimizes cost of production. The medical literature has thus far reported successful results from the of ACS injection in the treatment of OA.

With regards to knee OA, Baltzer et al. demonstrated that not only is ACS safe and effective in the treatment of knee OA, but its effects may persist for up to 2 years. The study was a prospective, randomized, double blind study of 376 individuals with knee OA. Patients were subjected to 6 injections of ACS over the course of 3 weeks. Clinical effects were assessed *via* WOMAC and VAS questionnaires at 7, 13, 26, and 104 weeks. Patients that received ACS reported significant improvements in their quality of life compared to the cohorts that received HA or placebo intra-articular injections over the span of the entire follow-up period^10^^,^^42^.

In another study, Yang et al. performed a prospective, randomized, double-blind experiment that evaluated 167 patients with knee OA to determine the effects of ACS versus saline solution intra-articular injections. Patients in the experimental group received 6 intra-articular injections of ACS over the course of 3 weeks. Joint evaluations were performed with VAS, WOMAC, Knee injury and Osteoarthritis Outcome Score (KOOS), and KSS clinical questionnaires at initial visit and then at 3, 6, 9, and 12 months follow-up. Statistically significant improvements were demonstrated at each follow-up period when compared to baseline levels^43^.

More recently, a prospective cohort study was performed to investigate the long-term effect of Orthokine injection on prevention of surgery for advanced stages of knee OA. The results of the study demonstrated that at 10 years follow-up, Orthokine was not able to significantly delay the need for knee arthroplasty when compared to a placebo control group^44^. However, the authors of this study did not take into account the use of other nonsurgical treatments previously performed on the patients in the study. In addition, no clinical or functional evaluations were performed. The sole parameter measured was incidence of knee arthroplasty following intra-articular injection of ACS. Therefore, we have no data describing the disease progression or symptomatology during the period between final injection and surgery. Furthermore, a study conducted by Baselga García-Escudero et al. demonstrated the effectiveness of ACS injections combined to physical therapy, showing positive results through the whole 2-years follow-up^45^.

Although the production of ACS increases the levels of naturally occurring IL-1 RA, the composition of the product may vary in concentration of cytokines and GFs among patients. Moreover, the direct effect of the serum injection on the metabolism of AC in knee OA has not yet been described^46^. In addition, the incubation process appears to stimulate the production of other cytokines and growth factors in whole blood, suggesting that the role of IL-1Ra in decelerating disease progression may be overestimated^47^.

Our study demonstrated results that are in line with current medical literature on the effects of ACS on knee OA. Direct comparison *via* statistical analysis was not able to be performed between the data from our study and the data in the medical literature on the same subject matter. This is due to differences in study protocol, primarily the follow-up period, the time frame of injections, as well as the number of injections performed between our study and the existing studies. Even so, the results obtained demonstrate a clear improvement in overall joint health.

Specifically, symptoms of knee OA showed a trend of alleviation evidenced by the change in VAS and WOMAC values between initial clinical evaluation and final follow-up at 6 months. For both these parameters, the difference was deemed statistically significant at *p* ≤ .05. It must be noted that not all the individual patients reflected this trend closely. This was seen more clearly in the VAS pain values reported. Two patients appeared to have increase in pain values after 1 week of therapy after initially showing signs of improvement since the 1st administration of ACS. The WOMAC also demonstrated the presence of a patient that did not necessarily follow the general trend. From the WOMAC box plot we can observe an increase in the amplitude of the interquartile boxes, suggesting a varied response to the treatment. That being said, the VAS and WOMAC were self-reported questionnaires and thus prone to errors. These values and observations must therefore be juxtaposed with the more objective KSS scores, which the orthopedic surgeon performed. In fact, if we look at the box plot for the KSS functional scores, we can observe a decrease in amplitude of the interquartile range boxes, as a result of a uniform positive response to the therapy. But in reality, the functional aspect of the KSS is largely reliant on the patient. It serves to give a brief functional assessment of the knee without having to perform objective tests. In a way, it is an abbreviated form of the WOMAC that serves as an adjunct to the clinical aspect of the KSS. Therefore, we must look at the KSS clinical scores to determine the extent of congruity between objective and subjective knee health. As shown in the box plot for KSS clinical scores, all the patients except for one demonstrate an increase in KSS clinical scores, indicating an improvement in overall function of the knee and hence health state of the joint.

## Limits

Although positive effects were demonstrated in our study, several limitations existed. Ideally, a larger sample size would have been preferred to avoid selection bias. Furthermore, as well as having a base comparison to demonstrate the extent of change in knee function, having a control group in the form of a placebo (with injection of saline), hyaluronic acid and/or PRP cohort would further elucidate the performance of ACS in contrast to current treatment modalities. Extended follow-up periods should be investigated to determine the extent as to which ACS can maintain adequate knee function and delay surgical knee replacement. However, the main purpose of this article was not to demonstrate the effectiveness of ACS therapy over the other treatments. The aim of this study is to observe the effects of Orthokine on the knee OA. Furthermore, given the composition of ACS following incubation, studies should attempt to determine which of the cytokines and growth factors that are found above baseline averages contribute to the amelioration of clinical and functional signs of knee OA. Such studies should attempt to isolate individual cytokines and growth factors and compare their effects individually.

## Conclusion

ACS represents the new direction of DMOADs for the treatment of knee OA by targeting the specific compounds responsible for the pathogenesis of disease. This therapy is a simple, safe, and conservative option for knee OA with limited adverse effects and few contraindications. It targets the main cytokine responsible for the inflammatory-degenerative cascade involved in OA, IL-1.

The use of ACS for the treatment of knee OA is supported by the results of our study, which correlate with existing studies in the medical literature. As such it has proven to be a viable alternative to the ever-expanding therapeutic options available to orthopedic surgeons when managing knee OA. As a future prospective it could be interesting to compare the effects of ACS with other biological therapies. For this reason, our next purpose is to conduct a controlled clinical study in order to clarify the role of ACS in the treatment of knee OA.

## Transparency

### Declaration of financial/other relationships

The authors declare no financial relationships. The peer reviewers on this manuscript have no relevant financial or other relationships to disclose.
